# Sediment distribution on the continental shelf in relation to stream inputs and contamination: hydrodynamic, chemical, mineralogical, and sedimentological characteristics (Ligurian Sea, Italy)

**DOI:** 10.1007/s11356-020-10259-4

**Published:** 2020-08-01

**Authors:** Laura Cutroneo, Cristina Carbone, Sirio Consani, Marco Capello

**Affiliations:** DISTAV, University of Genoa, 26 Corso Europa, I-16132 Genoa, Italy

**Keywords:** Sediment transport, Hydrodynamic, Stream input, Mineralogy, Metal concentrations, Continental shelf

## Abstract

River estuaries, continental shelf, and sediment contamination are closely linked from the point of view of sediment transport and diffusion that is governed by different factors such as sea waves and currents, river flows and floods, and sediment characteristics. Taking these factors into consideration, we have examined marine environmental and marine bottom sediments off the mouth of a stream to highlight the main ways of sediment and contaminant transport and diffusion on the continental shelf. For this purpose, we followed a multidisciplinary approach, studying circulation of water masses, hydrological characteristics of water column, distribution and main characteristics of sediment grain size, sediment mineralogical composition, and metal concentrations of bottom sediments. Our results allowed identifying the presence of preferential ways of sediment deposition and areas of sediment spread for the Entella Stream, as well as the origin of some metals.

## Introduction

Historically, cities and towns are concentrated along waterways and coasts with consequent alteration of the surrounding environment and input of contaminants in both fresh and marine waters. Contaminants in fresh water tend to accumulate in sediments, which are transported from rivers to the sea. Here, currents and rough seas spread and distribute sediments off the river mouth to the continental shelf and along the coasts (van der Oost et al., 2003). Sources of contaminants on rivers are multiple and contaminant concentrations of river sediments depend on the presence and distribution of human activities in all the catchment basin. At sea, the input of rivers is added to that of the activities directly present along the coasts and at sea (sewage drains, commercial ports and marinas, street runoff, shipyards, vessel traffic, fishing; Nicolau et al., 2006). In the case of metal contamination of marine sediments, factors that mostly control metal diffusion are the physical and chemical characteristics of sea water (temperature, salinity, and pH), the properties of sediments (grain size, chemistry, and mineralogy), and the dynamics of the area (Duran et al. 2012; Nieto et al. 2007).

Just because sediments play an important role in contaminant accumulation and transport, they are frequently used to identify contamination sources, study dispersion pathways and mechanisms, and determine the extent of the area involved in contamination and its time duration and evolution (Abdollahi et al. 2013; Alyazichi et al. 2016; Garcia-Orellana et al. 2011; Ruiz-Compean et al. 2017). However, several studies focus their attention only on a few specific abiotic characteristics of sediments (geochemistry or mineralogy) to trace back their origin and/or contamination degree (Ilgar and Sari 2008; Morillo et al. 2004; Shaari et al. 2015; Villaescusa-Celaya et al. 2000). On the contrary, other studies have shown that the use of multiple approaches is more useful in this research type to understand and highlight the different mechanics involved in the investigated area (Cutroneo et al. 2017; Martins et al. 2012).

Therefore, the goal of this paper is to characterise and follow sediments and metals transported by a stream on the continental shelf in front of its mouth using a multidisciplinary approach to understand sediment dynamics and identify any preferential ways of metal dispersion spreading associated with them. The use of the multidisciplinary approach, which takes in consideration the relationships between sediments (grain size and minero-chemical composition) and dynamics and hydrology (coastal currents and chemico-physical characteristics of water masses), allowed to define distribution, origin, and main transport directions of sediments and associated metal dispersion and highlight outliers in metal concentrations. The study area is the Gulf of Tigullio (Ligurian Sea, north-western Italy), a marine area where heavily populated stretches of coast are closely linked to areas of great natural value, such as the Marine Protected Area of Portofino, and where recent studies of sediment contamination highlighted the presence of outliers in metal distributions (Capello et al. 2016; Consani et al. 2019).

## Study area

**Fig. 1:**
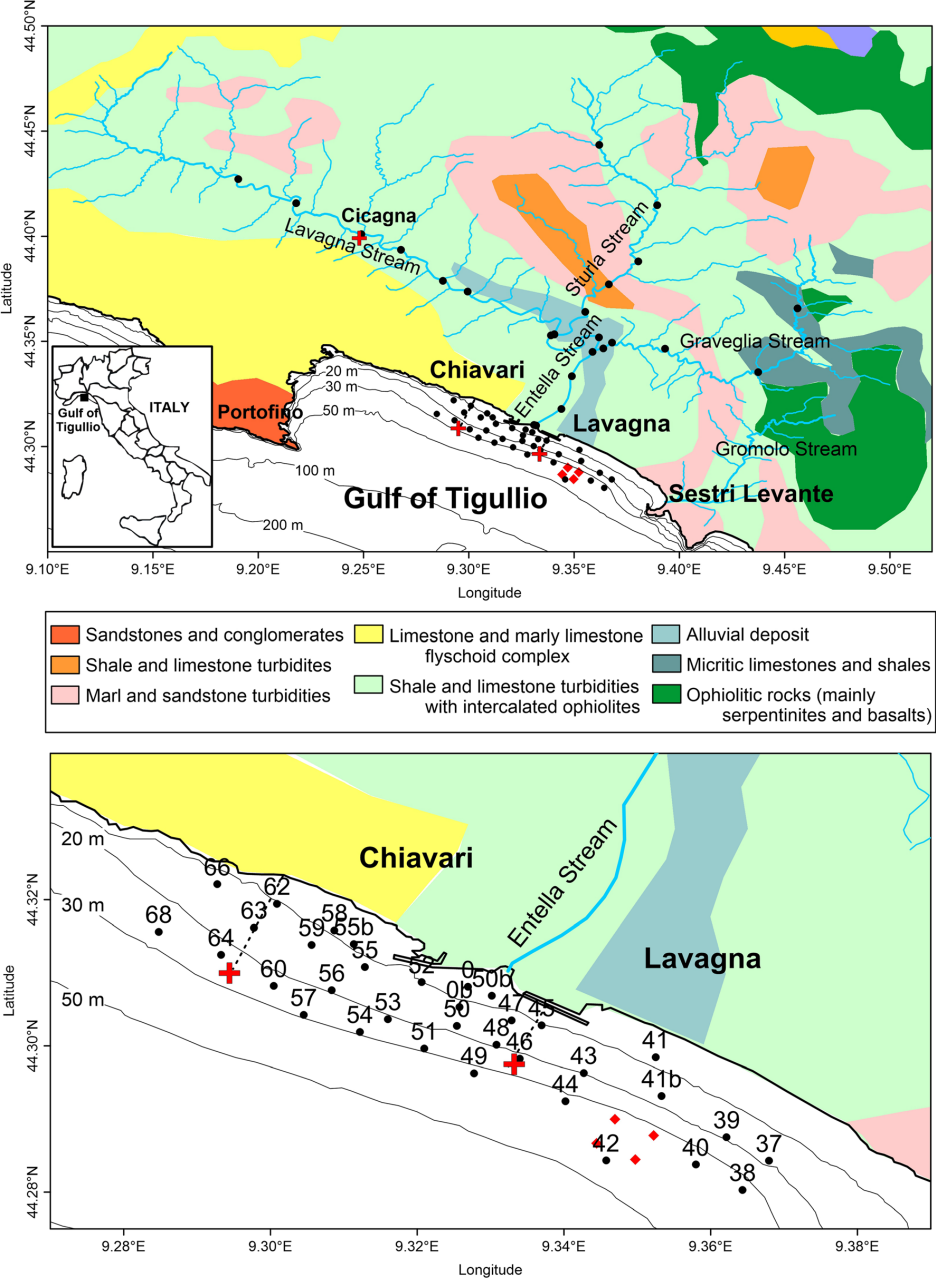
Location of the Gulf of Tigullio and the sampling stations (black dots) in the streams (Entella, Lavagna, Sturla, and Graveglia streams) and off the mouth of the Entella Stream, between 6 and 46 m depth. Red rhombuses identify a fish farm. The three red plus signs show sewage discharges of Cicagna (Lavagna Stream), Chiavari, and Lavagna (Gulf of Tigullio). Different colours of the background indicate the geolithological composition of the territory reported in the legend (ISPRA 2020)

The Gulf of Tigullio is in the eastern part of the Ligurian Sea (north-western Mediterranean Sea) and extends from the Portofino promontory to the headland of Sestri Levante (Fig. 1). The Entella Stream (8 km of length, 1700 mm year^−1^ of mean rainy influx, with peaks in October and November; Provincia di Genova 2009) flows at the middle of the gulf between the cities and the ports of Chiavari and Lavagna. The Entella Stream receives waters from three tributaries, the Lavagna, Sturla, and Graveglia streams, located in as many distinct valleys, forming a global catchment area of 370 km^2^.

Inland, especially along the Lavagna and Entella streams, there are some productive activities, such as production and processing of thermoplastic material, vegetable oils, pinwheels, electrical/electronic equipment, zinc oxide, junkyards, several quarries (limestone and slate), a landfill of solid urban waste and centres for the collection, and recovery of scrap metal materials.

Geologically, the Lavagna Stream (Fig. 1) crosses turbiditic shales and limestones (light green colour in Fig. 1) and, in a minor extent, limestone and marly limestone flyschoid complexes (yellow colour in Fig. 1). The Sturla Stream crosses the same turbiditic shales and limestones of the Lavagna Stream, marl and sandstone turbidites (pink colour in Fig. 1), and shale and limestones turbidites (orange colour in Fig. 1). The geology of the Graveglia basin is slightly different, as the stream also crosses an ophiolitic sequence (dark green in Fig. 1) formed by an oceanic basement (serpentinites, ophiolitic breccias, and basalts) and its related sedimentary cover of micritic limestones and shales (teal colour in Fig. 1). The total landslide is relatively greater for the Lavagna Stream where the landslide surface is 11% of the basin than for the Sturla and Graveglia basins that show a landslide surface of 9%. Consequently, the solid transport of the Entella Stream results heterogeneous for lithology and mean size of sediments. The Entella basin reports an average solid transport of 200 t year^−1^ km^−2^, which corresponds to a solid load of 35,000 m^3^ year^−1^ at the stream mouth (Regione Liguria 2011).

The shoreline of the study area (Fig. 1) is characterised by low and linear coasts produced by the alluvial deposits of the stream and is interrupted by the port breakwaters and beach groynes. The sea bottom is mainly sandy with a maximum depth of 50 m inside the gulf, and the bathymetries are parallel to the coast. Circulation of the gulf is predominantly westwards and characterised by inversion of only short time periods and presence of countercurrents along the coasts, as already highlighted in other areas of the Ligurian coast (Capello et al. 2014; Doglioli et al. 2004). Southern winds (SE and SW) dominate the general wave regime of the area, with the maximum wave height (7 m) registered with SW winds (libeccio) in autumn. For events with an annual return period, the wave breaking happens on a depth between 4 and 9 m for all directions of wave propagation (Regione Liguria 2011).

The Gulf of Tigullio (Fig. 1) has been and still is widely studied under different points of view, but researches on metal distribution in the area and mechanisms that regulate their diffusion have only recently carried out. In fact, sea water and sediment ecology (Albertelli et al. 1999; Capello et al. 2016; Licandro and Ibanez 2000; Misic and Covazzi Harriague 2008; Misic et al. 2016; Parravicini et al. 2013), coastal and sediment geology (Barsanti et al. 2003; Corradi et al. 2003; De Vita et al. 2012), and current dynamic (Capello et al. 2016; Doglioli et al. 2004) were deeply studied, but only Bertolotto et al. (2005) have addressed the problem of sediment contamination (only with three sampling stations off the Entella mouth) in a general characterisation of the sediments of the Ligurian coast and, recently, Capello et al. (2016) and Consani et al. (2019) have focus their attention on the metal input to marine bottom sediments from an abandoned mine in the eastern Gulf of Tigullio. The study of Consani et al. (2019) not only highlighted the presence in the coastal sediments of some metals clearly linked to the input deriving from the abandoned mine of Libiola and the geology of the area but also highlighted the presence of foreign metals of which the source is sought by the present study in the adjacent western area where another important stream for the Gulf is present (the Entella Stream).

## Materials and method

Marine sediment samples, hydrological characteristics, and currents were sampled and collected at 34 stations distributed between 6 and 46 m depth on the continental shelf off the mouth of the Entella Stream (Fig. 1) on 22 and 23 June 2016. About 500 mL of bottom sediments was sampled using a 5-L Van Veen grab at each station (Fig. 1) and then stored in new plastic jars rinsed with deionised water. Sediment samples were homogenised and then divided into three portions for the analyses of grain size distribution, mineralogical composition, and chemical content.

Grain size analysis of sediments was conducted following methodology described in Capello et al. (2016). Samples were wet sieved for grain size (Ø) > 63 mm (with 63, 125, 250, 500, 1000-μm steps for sand, and Ø > 2000 μm for gravel). The fine fraction (Ø < 63 mm) was analysed using a Coulter Counter® Multisizer 3 (Beckman Coulter, Inc.). Grain size results were expressed in percentage values of clay, fine silt, medium silt, coarse silt, very fine sand, fine sand, medium sand, coarse sand, and very coarse sand. Organic and inorganic sediment contents were measured. Sediments were combusted in an ISCO muffle (ISM320 mod.) at 550 °C for 3 h to remove the organic fraction (OF). The uncombusted fraction was weighed and used as the inorganic fraction (IF).

Powder X-ray diffraction (XRD) analysis with Co Kα radiation (current 20 mA, voltage 40 kV) was used for the mineralogical characterisation of 12 sediment samples distributed on two transects covering the entire study area: the first parallel to the coast (stations 0, 37, 39, 41b, 43, 55, 59, 63, and 66) and the second perpendicular to the coast off the mouth of the Entella Stream (stations 0, 50, 50b, and 51). XRD diagrams were collected on each dry sample (pre-grounded with a pestle) for 2*θ* (total scattering angle) between 5 and 80° at a scan rate of 5° min^−1^. Mineralogical results were expressed in percent, and quantification of minerals was performed according to the reference intensity ratio (RIR) method. Inductively coupled plasma mass spectrometry (ICP-MS) was used for determine fifteen metal (Al, Ca, Fe, Mg, Mn, As, Cd, Co, Cr, Cu, Ni, Pb, V, Zn, and Hg) concentrations in sediment samples (31 samples) according to methodology and standard quality assurance procedures reported in Capello et al. (2016).

A conductivity–temperature–depth (CTD) multiparametric probe (IdromarAmbiente) equipped with a turbidimeter (Turner Designs) was used to collect the hydrological data (temperature, conductivity, salinity, turbidity, and density of the water masses) in the water column. The turbidity of the water was expressed in formazin turbidity units (FTU, range 0–100 FTU), while the salinity was determined using the Practical Salinity Scale. A Teledyne RDI 600-kHz Workhorse® Vertical Acoustic Doppler Current Profile (V-ADCP) was used to collect current velocity and direction data in the water column at 29 of the 34 sampling stations. An external global positioning system (GPS) was used to collected navigational data for georeferencing data.

Distance (expressed in m) between sampling stations and the stream mouth was calculated to evaluate the effect of any dispersal of the material coming from the stream.

Fine fraction (< 63 μm) of stream sediments was sampled by sieving on the field and analysed in metal content and mineralogical composition to obtain a characterisation of what is transported by stream to the sea. Stream sediments were collected at 25 stations (Fig. 1) distributed along the Lavagna, Sturla, Graveglia, and Entella streams to cover the entire catchment basin. Stream samples were analysed with the same methodologies for metal and mineralogical analyses as marine sediments. The geology of the three stream valleys is quite variable, and results obtained from sediments of each stream were averaged but considered separately to highlight the single contribution of each sub-basin to marine sediments.

Principal components analysis (PCA), carried out with the free R software (v. 3.3.1; R Core Team 2020) using “Vegan” library, was used to identify patterns, groupings and outliers in the sampling station distributions in relation to metal concentration (concentrations of all fifteen metals analysed) and grain size characteristics (percentage of all grain size classes analysed) of sediments and distance between sampling stations and the stream mouth.

## Results and discussion

### Sediment input from the Entella basin

The mineralogical composition of sediments of the main sub-basins of the Entella Stream is reported in Table 1. Quartz is invariably the dominant phase. The sediments of the Lavagna, Graveglia, and Sturla streams showed a similar mineralogical composition, with phyllosilicates (chlorite and muscovite), calcite, and feldspars (plagioclase and k-feldspar) being the most present minerals after quartz. Compared with the Lavagna and Sturla streams, the Graveglia Stream sediments had a higher concentration of calcite, whereas phyllosilicate and feldspar concentrations decreased. Moreover, minerals related to the ophiolitic rocks outcropping in the area, such as talc and serpentine, constituted almost the 10% of the Graveglia Stream sediments.

**Table 1:** Mean mineralogical composition (in %) of sediments of the main streams of the Entella basin. Number of samples for each stream used to calculate mean and standard deviation of minerals are as follows: 9 samples for Lavagna Stream, 6 samples for Sturla Stream, 5 for Graveglia Stream, and 5 for Entella Stream. To simplify the table, values corresponding to 0 have not been indicated and the cell has been left purposely empty

Mean ± standard deviation	Lavagna Stream	Sturla Stream	Graveglia Stream	Entella Stream
Quartz	33.8 ± 4.8	37.7 ± 4.0	34.9 ± 6.4	43.5 ± 3.4
Plagioclase	17.9 ± 2.7	14.5 ± 6.7	12.4 ± 3.2	18.6 ± 4.9
Calcite	3.9 ± 1.9	11.7 ± 9.8	26.5 ± 16.2	3.0 ± 0.7
Chlorite	27.4 ± 3.2	23.3 ± 5.8	13.0 ± 3.3	23.7 ± 3.6
Muscovite	12.4 ± 2.2	11.5 ± 3.6	5.4 ± 3.1	9.6 ± 2.9
K-feldspar	4.6 ± 3.4	1.3 ± 2.0		1.6 ± 1.8
Talc			2.4 ± 3.4	
Serpentine			5.4 ± 2.3	

The bulk chemistry of the stream sediments (Table 2) reflected the shift observed in the mineralogical composition. The Graveglia Stream sediments had a doubled mean concentration for Mg, Co, Cr, and Ni compared with the other stream sediments, and the increase of calcite led to a correspondent increasing of Ca. Standard deviations for metals were higher than those observed for the other streams, probably because the geology of the Graveglia Basin abruptly changed. In fact, ophiolitic rocks outcropped mainly in the upper part of the basin, whereas in the lower part, limestones were dominant (Fig. 1). On the contrary, Al concentration was higher in the stream sediments of the Lavagna and Sturla basins, as a consequence of the higher abundance of silicates (Fig. 1). All other considered elements did not show any significant variation, with the only exception of Mn, whose concentration in the Sturla Stream sediments was remarkably lower compared with the other streams.

**Table 2:** Mean concentration (± standard deviation) of metals found in the stream sediment samples

Mean ± standard deviation	Lavagna Stream	Sturla Stream	Graveglia Stream	Entella Stream
Al (%)	2.08 ± 0.12	1.85 ± 0.35	1.38 ± 0.47	2.10 ± 0.15
Ca (%)	2.41 ± 0.72	4.40 ± 2.18	9.92 ± 6.46	2.15 ± 0.26
Fe (%)	3.79 ± 0.23	3.23 ± 0.64	2.55 ± 1.12	3.89 ± 0.36
Mg (%)	1.03 ± 0.07	1.14 ± 0.20	2.04 ± 0.93	1.46 ± 0.48
Mn (ppm)	1000 ± 194	625 ± 388	1014 ± 363	1121 ± 318
As (ppm)	5.42 ± 0.72	3.90 ± 0.40	3.08 ± 1.24	5.58 ± 0.93
Cd (ppm)	0.26 ± 0.06	0.31 ± 0.12	0.23 ± 0.09	0.29 ± 0.10
Co (ppm)	19.5 ± 1.7	18.7 ± 5.2	33.5 ± 16.9	22.9 ± 3.0
Cr (ppm)	53.3 ± 6.5	73.5 ± 14.9	186.0 ± 89.0	93.7 ± 44.4
Cu (ppm)	75.6 ± 8.0	62.2 ± 16.1	75.2 ± 21.8	82.0 ± 10.5
Ni (ppm)	74.6 ± 6.6	89.0 ± 14.6	236.0 ± 121.0	119.0 ± 36.1
Pb (ppm)	41.9 ± 5.17	41.1 ± 11.8	33.8 ± 20.4	42.2 ± 3.2
V (ppm)	23.9 ± 1.6	26.5 ± 5.5	28.7 ± 12.1	31.0 ± 7.0
Zn (ppm)	140.0 ± 16.1	109.0 ± 20.1	120.0 ± 67.0	206.0 ± 18.9
Hg (ppb)	104.0 ± 33.4	87.0 ± 22.0	68.0 ± 26.6	100.0 ± 33.6

For most metals, sediments of the Entella Stream displayed characteristics like those of Lavagna and Sturla streams, which supply the major part of the total sediments. The influence of the Graveglia Stream could be observed only for Co, Cr, Mg, and Ni, which remained higher compared with the other sediments. The Entella Stream sediments showed enrichment in Zn which is not present in the other sediments. Zn, widely used in many productions from industrial metallurgy to the production of personal care products, could be influenced by city discharges and metal waste materials deposits that are located along the Entella banks.

### Marine environment and marine sediments

**Fig. 2:**
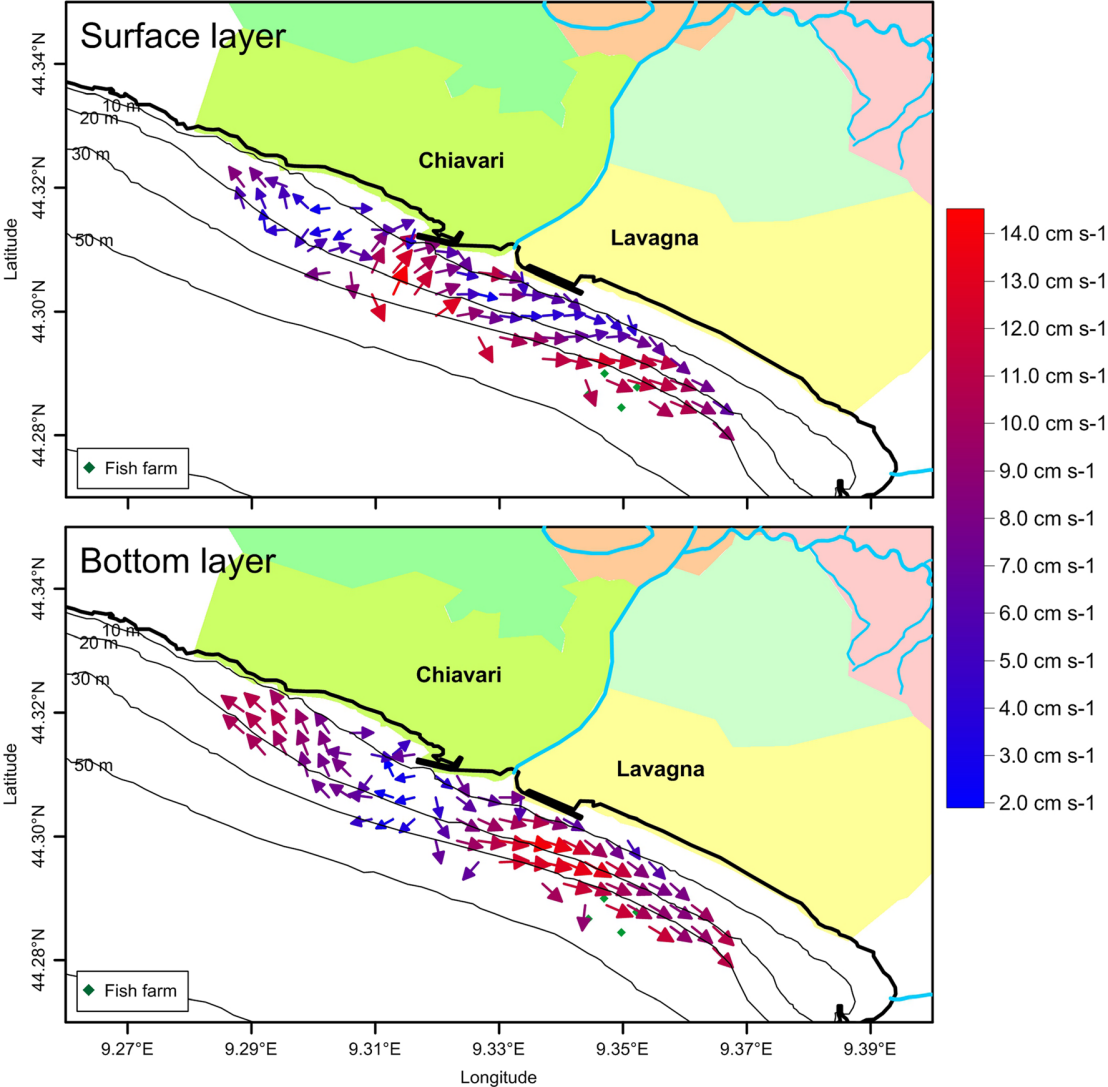
Current vector distributions in the surface (above) and bottom (below) layers of the study area

In the water column and especially in the bottom layer, sea currents (Fig. 2) were divided into two distinct zones separated by the Entella Stream mouth and a central water corridor. East of the stream mouth, currents flowed eastwards according to the coastal countercurrents that is a typical situation of the Ligurian coasts as already found by Capello et al. (2014) along the western Ligurian coast. On the contrary, west of the mouth, currents flowed westwards. Current velocities were between 1.5 and 15.0 cm s^−1^ in the surface and bottom layers, respectively, highlighting a calm condition, according to the weather that characterised the monitoring days (calm sea and no wind).

**Fig. 3:**
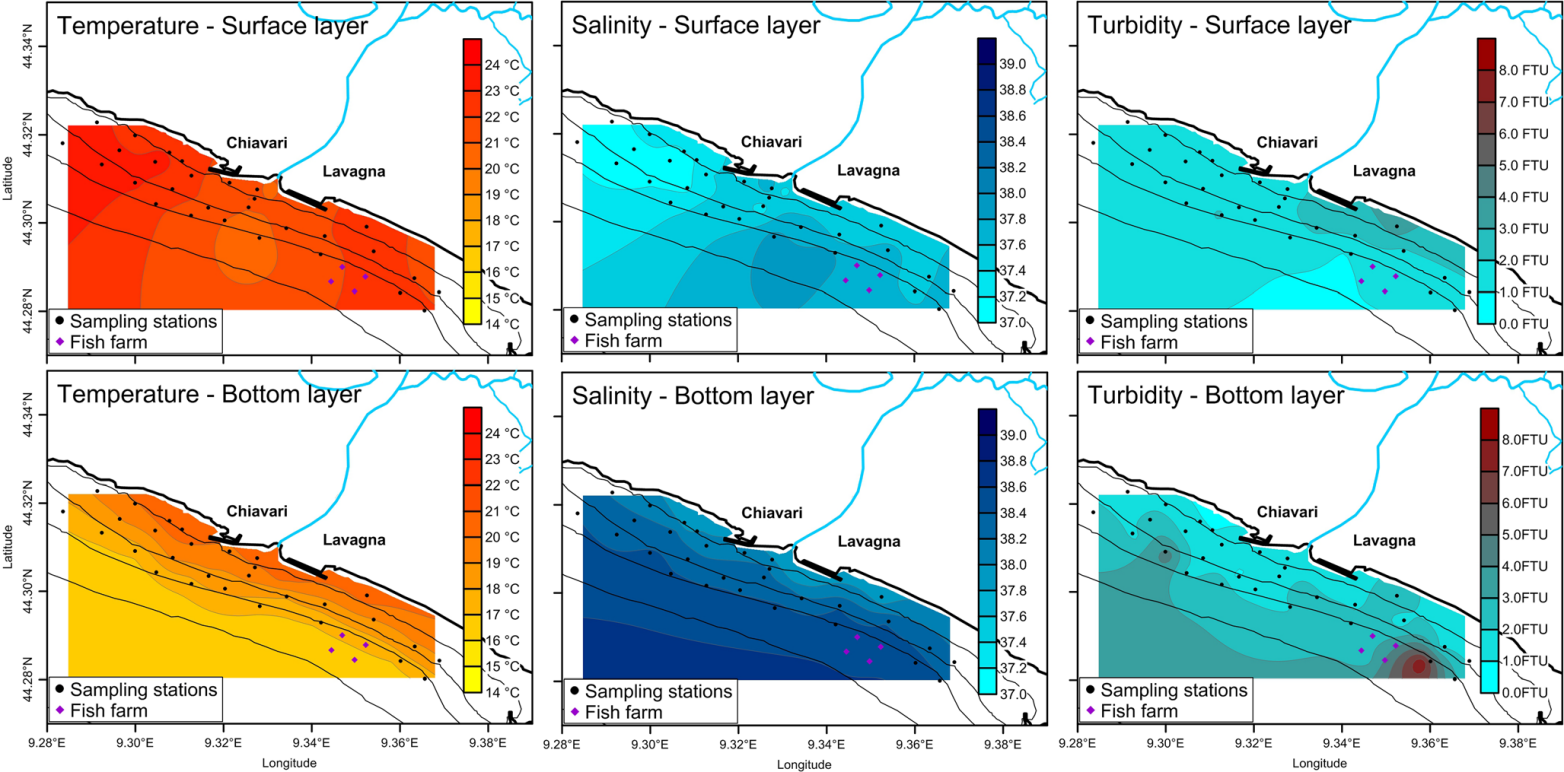
Horizontal distributions of temperature (°C, left), salinity (centre), and turbidity (FTU, right) in the surface (above) and bottom (below) layers

Water column was stratified according to the seasonal trend, showing higher temperatures and lower salinity in the surface layer than in the bottom one (Fig. 3). In the surface layer, temperatures showed relatively lower values off the stream mouth than in the rest of the area, highlighting the presence of reduced stream input of fresh water due to the few rains that fell during the days before the measures. On the bottom layer, temperature and salinity tend to be distributed according to the bathymetry, showing the effect of the season and confirming the sea calm situation. Water turbidity did not show any differences in both the area and the water column, corroborating the low input of freshwater and solids derived from the stream in accordance with the period of the year (early summer; Provincia di Genova 2009) in which the sampling took place.

The organic content of bottom sediments was relatively low and always < 3% in all the samples showing a uniformity of the area from this point of view.

**Fig. 4:**
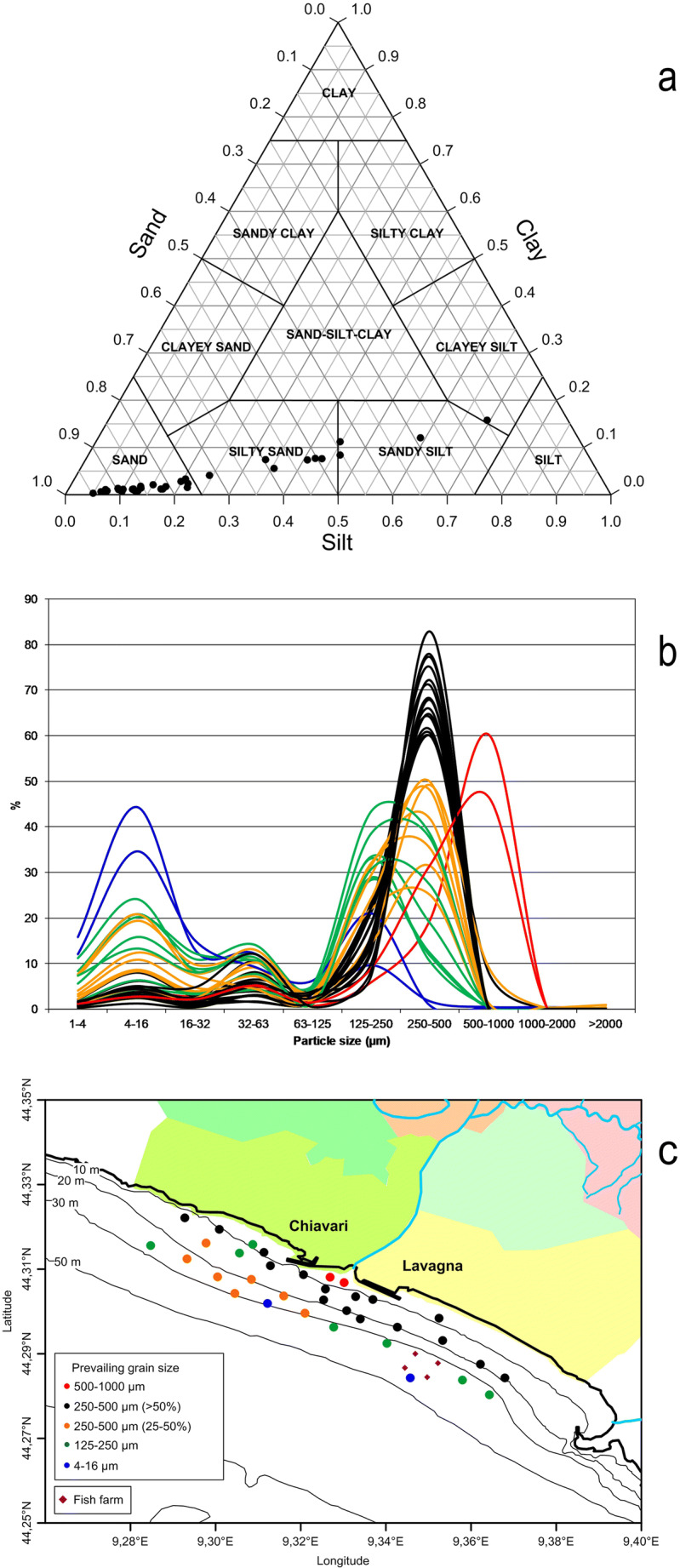
**a** The Shepard’s triangle diagram for the sediment samples. **b** Distribution of sediment grain size (in %): curves with different mode corresponding to a specific granulometric class are highlighted with different colours: blue—prevalence of 4–16 μm (> 30%); green—prevalence of 125–250 μm (25–50%); black—prevalence of the 250–500 μm class (> 50%); orange—prevalence of 250–500 μm (25–50%); red—prevalence of 500–1000 μm (> 40%). **c** Distribution of the sediment grain size classes in the study area

As resumed by the Shepard’s triangle diagram (Shepard 1954) shown in Fig. 4a, sediments lie within a sand and silt field, with most of the samples composed of sandy material and only two samples made of very fine material.

From the grain size distribution curves (Fig. 4b), five groups of stations can be distinguished based on the prevalence of a given particle size class, despite the presence of two or three modes in some cases: blue, prevalence of 4–16 μm (> 30%); green, prevalence of 125–250 μm (between 25 and 50%); black, predominance of the 250–500 μm class (> 50%); orange, prevalence of 250–500 μm (25–50%); and red, prevalence of 500–1000 μm (> 40%).

Following the subdivision into 5 prevalent granulometric classes found, sediment dimensions tended to be arranged according to the bathymetries with particle size generally decreasing from coarse to fine from coast to open sea (Fig. 4c). In particular, the two samples at the mouth of the Entella Stream (red samples: stations 0 and 0b) showed the prevalence of material with dimensions of coarse sand (between 500 and 1000 μm), whereas the other samples were mostly made of sand or silty sand.

Sediment size decrease from coast to open sea is mainly evident and regular in front of the coast of Lavagna, where black, green, and blue samples were spread on a bigger area and followed regularly bathymetries and the coast linearity. Off the Chiavari coast, the size distribution is more complicated and characterised by the prevalence of orange samples (25–50% prevalence of 250–500 μm) and the mixed presence of black and green samples in a narrow coastal strip (within 15 m depth). Therefore, from the point of view of particle size, two different areas can be highlighted: one east of the stream mouth where the medium sand is transported and one west of the stream mouth where transport of fine sediment prevails.

According to this size distribution, sediment samples were poorly sorted (56%) or very poorly sorted (32%), with only the remaining 12% moderately sorted, which correspond to a probable deposition close to the source, a fast deposition, a diffused wave action, a concentrated deposition in the area, or an excessive volume of sediment (e.g. during floods or storm) (Baiyegunhi et al. 2017; Watson et al. 2013). The poorly sorting of sediments were also proved by the presence of more than a mode in the grain size distribution in 44% of samples, as shown in Fig. 4b.

**Fig. 5:**
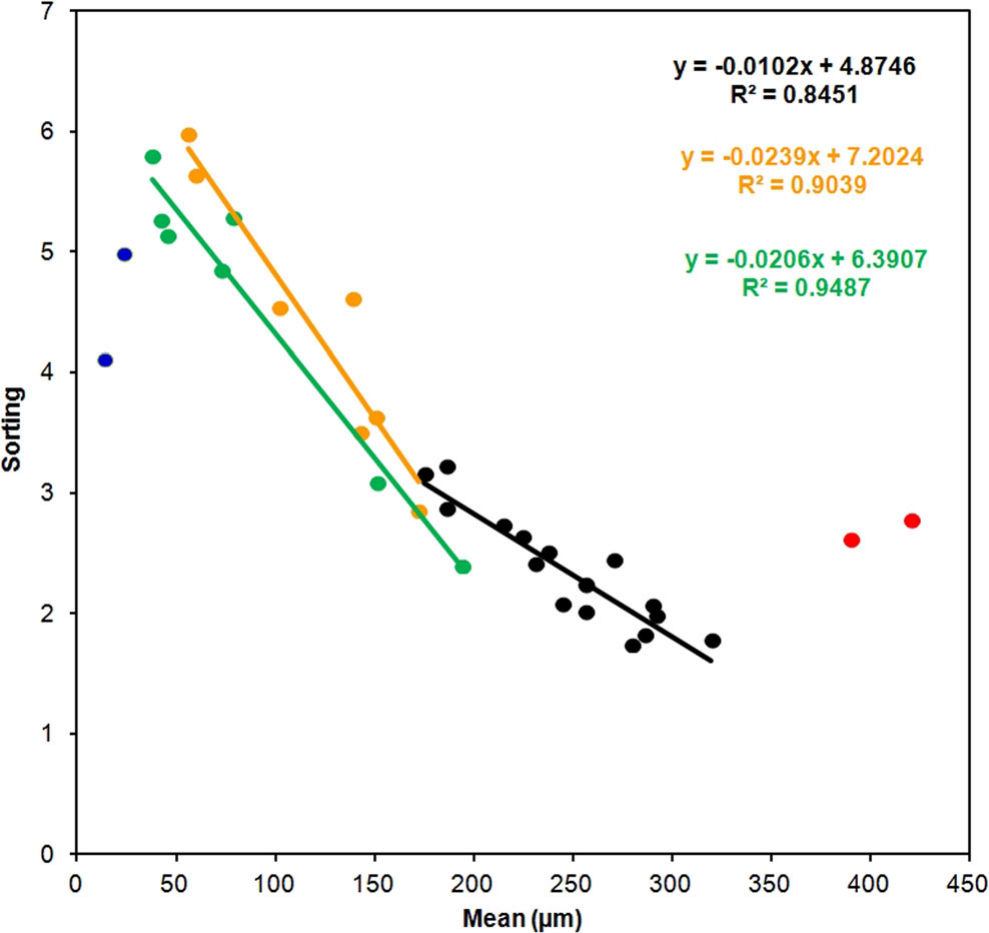
Plots of mean grain size against sorting for the 34 sediment samples studied

Station groups determined by the sediment grain size distribution were visible also by the sorting plot showed in Fig. 5 and obtained following Watson et al. (2013). Figure 5 highlights the presence of pronounced shifts between the groups. A negative correlation was observed between sediment grain size and sorting in all sample groups, with the exceptions of the groups showed in both red and blue that are composed by only two samples each. These results meant that the samples with the best sorting were those with the biggest sediment size (red samples) and, consequently, those taken in the most dynamic environments (the Entella mouth) and those with the smallest size (blue samples) sampled at the bottom band over 30 m depth. The goodness of the equations (*R*^2^ > 0.84) demonstrated the validity of these results.

Mineralogical results are reported in Table 3. The main minerals found in all the samples were quartz (ranging between 43 and 60%), plagioclase (11–25%), chlorite (11–20%), calcite (2–15%), and muscovite (4–7%), with smaller quantities of serpentine and clinopyroxene (2–4%), and, only in a few stations, amphibole (2–3%) and talc (1%). Mineralogical composition of sediments reflected the composition of the outcropping rocks of the Entella basin. Sedimentary rocks (shales, limestones, and sandstones; Fig. 1) could be considered the main sources of plagioclase, chlorite, calcite, and muscovite. These phases could be related to the input of the Sturla and Lavagna streams. Ophiolitic rocks, which occurred only in a smaller part of one of the tributaries of the Entella Stream, the Graveglia Stream (Fig. 1), could account for the presence of serpentine, clinopyroxene, amphiboles, and talc.

**Table 3:** Mineralogical composition of sediment samples. Values are expressed in percent. To simplify the table, values corresponding to 0 have not been indicated and the cell has been left purposely empty

Station	0	37	39	41b	43	50	50b	51	55	59	63	66
Quartz	56	54	57	57	60	45	55	56	45	56	51	43
Chlorite	18	11	11	13	15	16	18	20	15	17	17	13
Plagioclase	13	14	21	15	12	24	11	11	25	13	13	19
Calcite	2	5	3	3	4	8	3	2	5	4	8	15
Muscovite	6	6	5	6	6	5	6	7	5	6	5	4
Serpentine	2	4	3	3	3	2	3	2	2	2	3	3
Clinopyroxene		3		3			4	2	3	2	2	3
Amphibole	3	2										
Talc		1									1	

These results compare with results reported by Capello et al. (2017) in the easternmost part of the Gulf of Tigullio. However, some differences were found in comparison with sediments of the easternmost gulf area: sediments of the study area were richer in quartz, and the concentration of minerals related to ophiolitic rocks was lower. This fact could reflect the higher volumetric importance of ophiolitic rocks in the Gromolo Stream basin (Fig. 1), the watercourse which supplies the main sediment input in the easternmost part of the Gulf of Tigullio (Abbate et al., 1980).

Concentration ranges for each considered metal found in the superficial bottom sediments are reported in Table 4. These values were comparable to those found by Bertolotto et al. (2005) in three superficial sediment samples collected in the area in 2005 (in front of the Entella Stream mouth) highlighting a stationary state of the sediment composition.

**Table 4:** Statistical descriptive parameters (minimum, maximum, mean, median, and standard deviation) of metal concentrations found in marine sediment samples. Values exceeding the Italian legal limits are in italics (Italian Ministerial Decree 56/2009)

	Minimum	Maximum	Mean	Median	Standard deviation
Al (%)	1.30	1.92	1.70	1.70	0.13
Ca (%)	1.70	8.31	2.63	2.34	1.18
Fe (%)	2.77	4.69	3.43	3.35	0.33
Mg (%)	1.83	3.48	2.44	2.35	0.43
Mn (ppm)	663	1134	896	906	115
As (ppm)	6.2	*20.7*	*14.1*	*13.5*	3.98
Cd (ppm)	0.02	0.11	0.06	0.06	0.02
Co (ppm)	16.7	25.1	21.3	21.6	1.49
Cr (ppm)	*97*	*284*	*151*	*143*	39
Cu (ppm)	19.3	63.9	33.4	30.8	10.4
Ni (ppm)	*117*	*243*	*164*	*154*	32
Pb (ppm)	14.0	*31.6*	21.7	20.7	4.5
V (ppm)	32	100	40	36	13
Zn (ppm)	64	119	91	91	13
Hg (ppb)	8.0	124.0	31.8	23.0	23.9

**Fig. 6:**
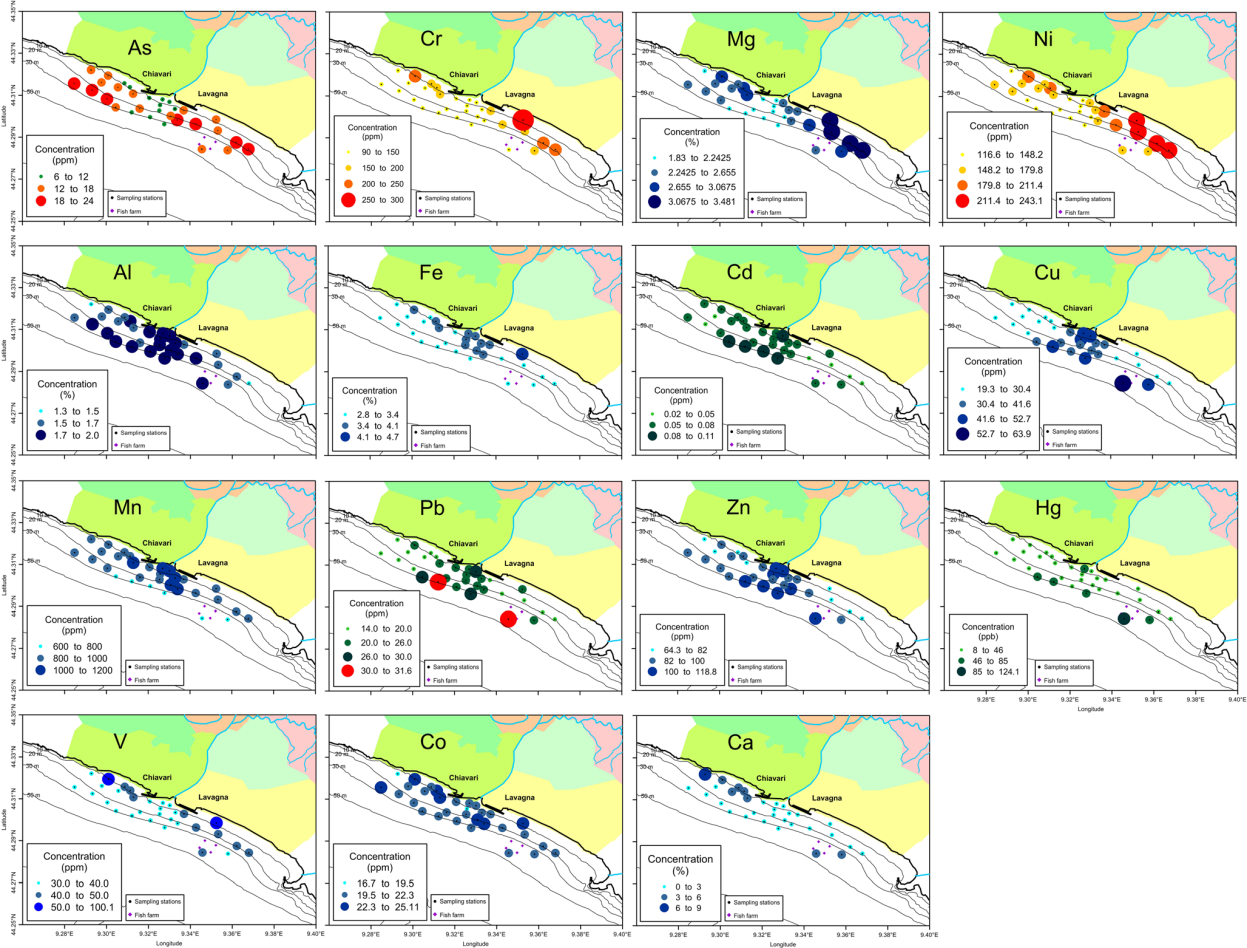
Metal distribution in the bottom sediments of the study area. Values are expressed in parts per million for As, Cd, Co, Cu, Cr, Mn, Ni, Pb, V, and Zn; in percent for Al, Ca, Fe, and Mg; and in parts per billion for Hg. Green and red scale colours refer to metals that have a concentration limit for the Italian law (green: under the limit; from yellow to red: over the limit; Italian Ministerial Decree 56/2009), while blue scale colours refer to metal without law limit

Metal distribution (Fig. 6), unlike mineralogical composition, was not homogeneous. The E–W subdivision seen in the currents and sediment grain size can also be identified in the metal distribution: in fact, some metals (As, Cr, Mg, V, and Ni) presented minimum concentrations in the central sector (inside the “dividing corridor” created by the water of the Entella Stream) and higher values on the two sides, whereas other metals (Al, Fe, Mn, Cd, Cu, Pb, and Zn) were distributed in the opposite way and had higher concentrations in the corridor and lower values on the two sides (only with a few exceptions). Consequently, the first metal group could not directly derive from the Entella Stream but had other sources, whereas the latter metals seem to come directly from the Entella input. Overall, the stream waters and its solid inputs act as watersheds along the Tigullio coast.

These different metal distributions were also confirmed by the Pearson’s correlation coefficient matrix (Table 5), which allowed some consideration on the sediment provenience. Mg, Cr, and Ni were strongly positively correlated (coefficient always > 0.9), and their maxima invariably lied in the eastern zone, where minerals related to ophiolitic rocks were most abundant. In fact, it is well known that these metals are typical tracers of sediments deriving from such rocks (Dey et al. 2018; Salmanpour et al. 2017). From this, it is possible to deduce that the distribution of Mg, Cr, and Ni in the superficial bottom sediments is mainly controlled by the input of the Gromolo Stream due to the geology of its catchment basin and the Libiola mine waters that it receives and brings to the sea (Consani et al., 2017). The origin of these elements is mainly geological; therefore, even if their concentration exceeded the threshold set by the Italian Law (Italian Ministerial Decree 56/2009) as reported in Table 4, they did not represent a “real” contamination.

**Table 5:** Pearson’s correlation coefficients among metal concentrations in the marine sediments. Observation number: 31. Strong positive correlations (*p* value = 0.001) are in italics

	Al	Ca	Fe	Mg	Mn	As	Cd	Co	Cr	Cu	Ni	Pb	V	Zn	Hg
Al	1.00														
Ca	− 0.62	1.00													
Fe	0.25	− 0.28	1.00												
Mg	− 0.59	0.06	0.16	1.00											
Mn	− 0.05	− 0.24	0.40	0.04	1.00										
As	− 0.54	0.08	− 0.22	*0.58*	0.01	1.00									
Cd	*0.63*	− 0.05	0.23	− 0.66	− 0.21	− 0.66	1.00								
Co	0.16	− 0.45	*0.67*	0.43	0.34	0.30	0.00	1.00							
Cr	− 0.53	0.11	*0.49*	*0.91*	0.15	0.45	− 0.51	*0.55*	1.00						
Cu	*0.71*	− 0.23	0.10	− 0.52	− 0.26	− 0.68	*0.63*	− 0.16	− 0.46	1.00					
Ni	− 0.57	0.06	0.25	*0.98*	0.08	*0.50*	− 0.64	0.43	*0.94*	− 0.49	1.00				
Pb	*0.72*	− 0.30	0.38	− 0.45	− 0.21	− 0.55	*0.74*	0.13	− 0.30	*0.87*	− 0.42	1.00			
V	− 0.32	0.15	*0.72*	*0.57*	0.05	0.23	− 0.15	*0.54*	*0.83*	− 0.29	*0.64*	0.00	1.00		
Zn	*0.83*	− 0.47	0.21	− 0.63	0.10	− 0.60	*0.69*	0.08	− 0.57	*0.79*	− 0.62	*0.78*	− 0.40	1.00	
Hg	*0.52*	− 0.03	− 0.09	− 0.35	− 0.50	− 0.36	*0.52*	− 0.15	− 0.36	*0.85*	− 0.37	*0.72*	− 0.22	*0.54*	1.00

Al, Cd, Cu, Pb, Zn, and Hg could be clustered together (Table 5). Al is usually found in silicates, such as feldspars and phyllosilicates (Bibi et al., 2016), and it is reasonable to assume that it and the other metals of its cluster were controlled by these silicates transported mainly by the Entella Stream. A possible explanation for the enrichment in Cu, Zn, Cd, and Pb of the sediments correlated not only to the input of the Entella Stream could be the presence of many sulphide (mainly Cu and Zn sulphides) mining areas in the basin (Zaccarini and Garuti 2008) but also to the city discharges, landfills of waste, and deposits of metallic materials in the Entella Stream basin. Hg was not uniformly spread in the area and showed relatively high values off the Entella mouth not showing clear clues about their original input, even if its presence could be mainly due to human activities (Gagnon et al. 1997). V is distributed on the sides of Entella mouth with maxima along the coast, and it was correlated with metals of the Mg group and with Fe. This double correlation could be explained with the fact that V often substitutes Fe^3+^ in minerals such as for example chlorites (Bailey 1988), but it is also present in minerals from ultrabasic rocks, such as amphiboles (Hawthorne et al. 2012) that are present in the study area (Table 3). Co was the other metal correlated to Fe, and both metals showed a quite uniform distribution. Fe showed only slight variations between sampling station and a slight increase off the mouth of the Entella Stream and along the coasts, especially at station 41, where it had its maximum. A brief remark can be made on station 41, which showed the highest concentrations of many of metals distributed at the two sides of the stream mouth (Co, Cr, Fe, Mg, Ni, V), signalling the presence of an additional metal source that could be represented by the Port of Lavagna, located upstream this station following the coastal countercurrent.

As was poorly correlated with Ni, while Ca and Mn did not show any positively correlation with other metals. Mn showed a slight tendency to be concentrated in sediments near the mouth of the Entella Stream, whereas As had an marked opposite trend (Fig. 6). The slight enrichment of Mn could be related to the presence of numerous mining sites in the rocks outcropping in the Entella basin (Cabella et al. 1998).

Metal distributions confirmed what already was found by Capello et al. (2016, 2017), who identified many different sources for metals in the superficial bottom sediments collected in an adjacent area. The mineralogical composition of the sediments obtained through XRD was not able to explain alone the distribution of metals. The only difference in metal distribution explained by mineralogy was the relatively high concentration of Mg, Ni, and Cr, which was related with an increase of minerals from ophiolitic rocks. Other factors should be invoked to explain the distribution of metals in the sediments.

The eastward principal transport of the sediments could account for the relatively high concentration of some elements (Cr, Mn, Pb, and V) observed in the sediments of the eastern part of the Tigullio Gulf by Capello et al. (2016) and Consani et al. (2019).

**Fig. 7:**
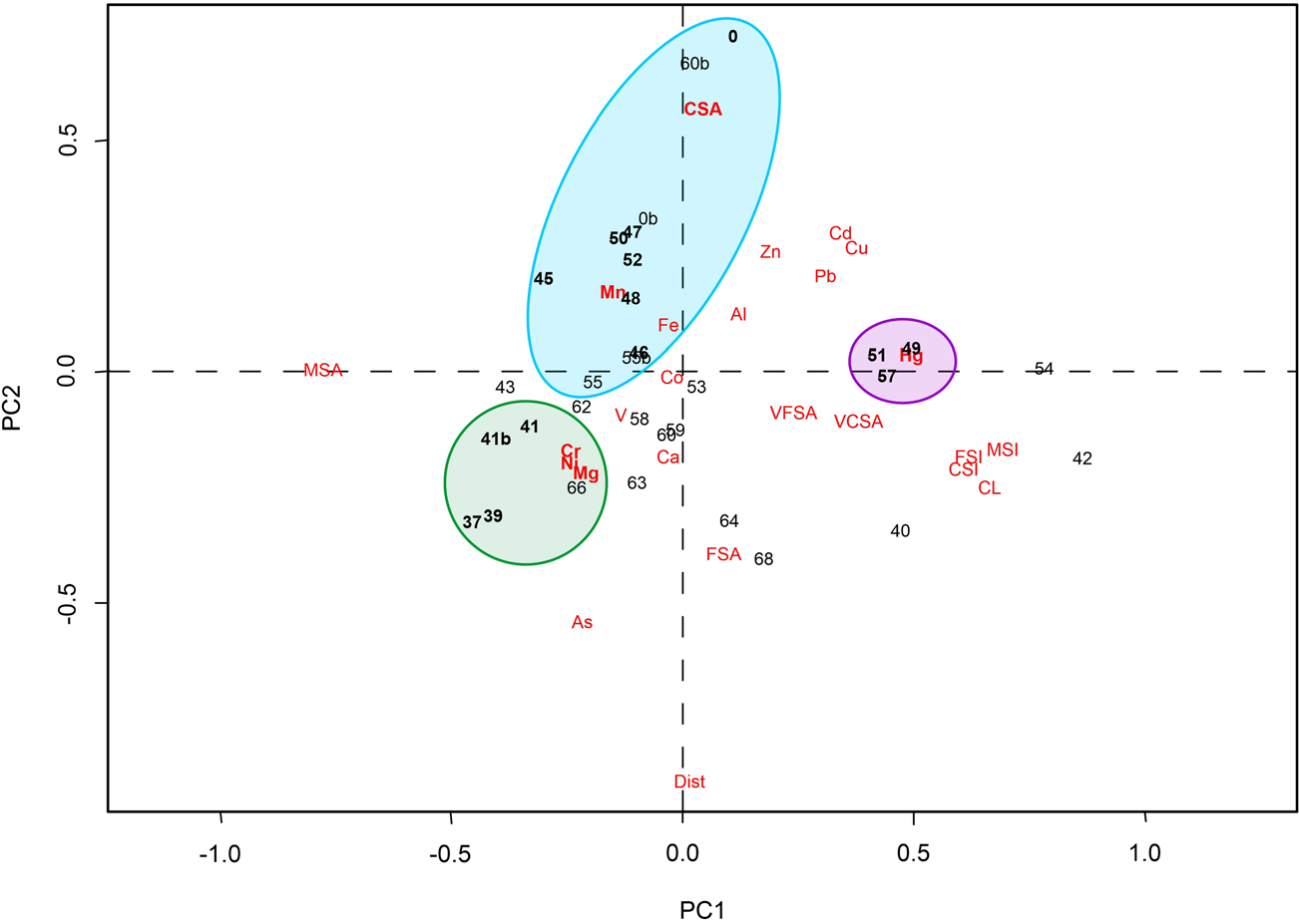
Results of principal component analysis based on the chemical and physical characteristics of sediment samples. Dist distance of sampling station from the stream mouth, CL clay, FSI fine silt, MSI mean silt, CSI coarse silt, VFSA very fine sand, FSA fine sand, MSA mean sand, CSA coarse sand, VCSA very coarse sand

In order to test if the hypothesis formulated based on so many variables were correct, principal component analysis was performed (Fig. 7). PCA confirmed that the grain size distribution followed the sea bottom bathymetry; in fact, distance of sampling station from the stream mouth was positively linked with fine sediments and negatively linked to the coarse sediments. Distance from the mouth also showed positive correlation with some metals such as As, Mg, Ni, and Cr that were particularly concentrated at the sides of the mouth and negative correlation with Cd, Cu, Fe, Mn, Zn, and Pb that were more concentrated in front of the mouth. The PCA confirmed also the indications on the different distribution of metals observed. The sampling points eastward of the Entella Stream mouth (37, 39, 41, and 41b, highlighted with a green circle in Fig. 7) can be distinguished from the others for their Cr, Ni, and Mg contents, confirming that the main difference between these sediments and the others is the increased importance of the contribution of ophiolitic rocks. The sampling points near the Entella mouth (0, 46, 47, 48, 50, and 52, highlighted with a blue circle in Fig. 7) are characterised by the presence of coarse sediments and Mn. Hg showed a correlation in those stations (49, 51, and 57, highlighted with a violet circle in Fig. 7) offshore but located off the mouth of the Entella Stream.

**Fig. 8:**
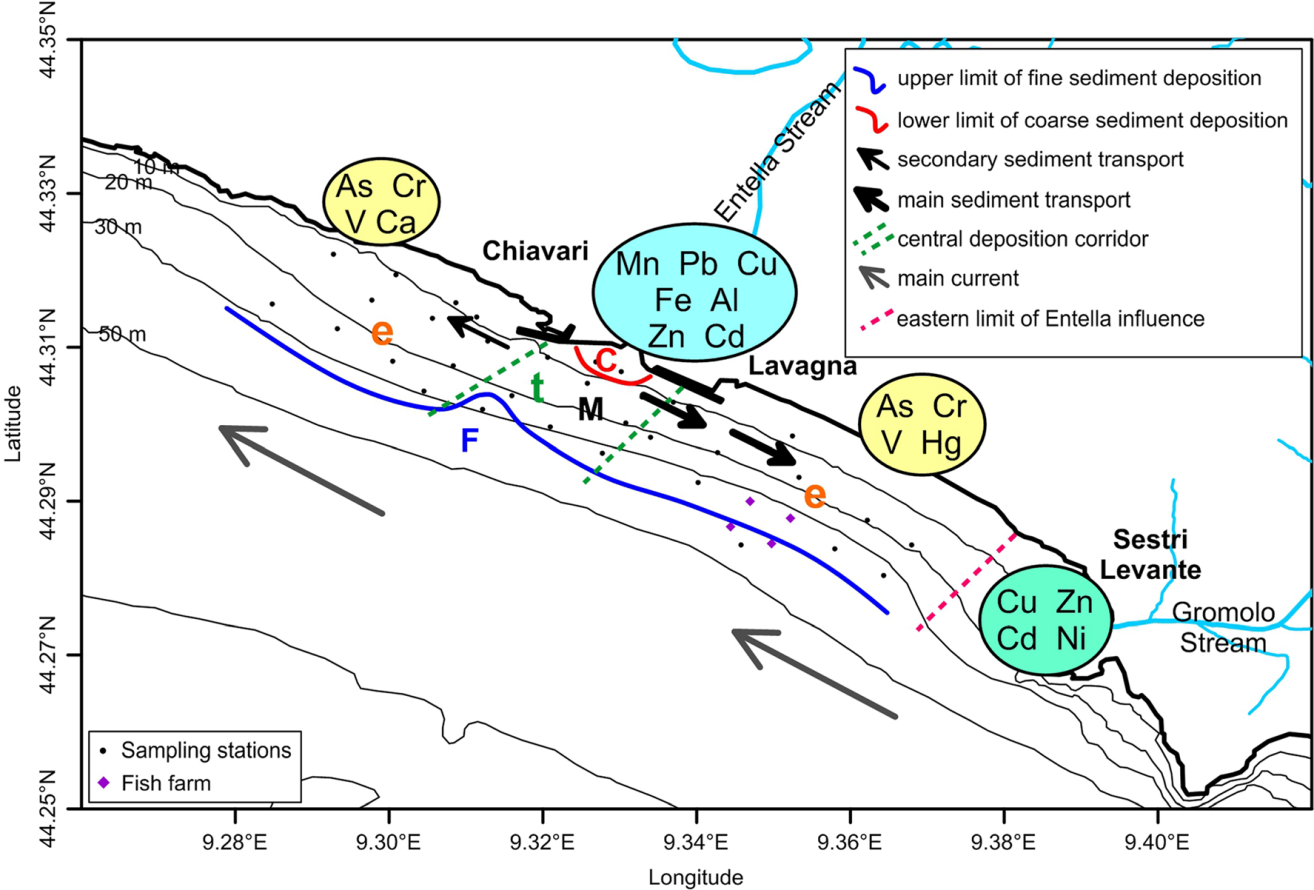
Schematic summary of sediment transport directions in the study area. Grey arrows show the main circulation of the Ligurian Sea; black arrows and black bold arrows show the preferential ways of sediment transport from the stream; green dotted line shows the first deposition area of stream material in front of the mouth (t); red dotted line indicates the eastern limit of the influence of the Entella sediments; red and blue lines highlight the off-shore limit of the deposition area of coarse and fine sediments, respectively. C: coarse sediments; M: mean-size sediments; F: fine sediments; t: components deriving from the stream; e: external components. In the coloured circles, distribution of metals depending on their origin: metals deriving from the Entella basin in the light-blue circle, from the Gromolo basin in the green circle, and metals with other origin in the yellow circles

Combining the results provided by all different analysis carried out in this study and according to results found by Capello et al. (2016) and Consani et al. (2019), a summary of the main characteristics of sediment and metal transport and diffusion on the continental shelf off the eastern part of the Gulf of Tigullio has been developed (Fig. 8) and reported below:

- An east–west division of the study area is produced by the presence of a NE–SW oriented deposition corridor in front of the mouth of the stream.
- The main sediment transport is eastward, whereas the secondary sediment transport is westward.
- The grain size distribution of sediments follows the bathymetry and the influence of stream discharges.
- The first deposition of the main mineralogical and chemical components of the stream basin is in the NE–SW oriented corridor in the central part of the area.
- The distribution of the external stream components is in the east and west sides of the central corridor.

## Conclusions

Our results showed that peculiarities on the distribution and diffusion of sediments and related metals of the Entella Stream on the continental shelf can be identified, such as the presence of preferential areas of sediment deposition or areas of sediment spread. Connected to these, areas of preferential accumulation of metal can be identified and, in some cases, they are closely linked to the contributions of the stream. It is hard to unravel the specific origin of those metals directly related to the Entella Stream sedimentary input, such as Cd, Cu, Hg, Pb, and Zn. In fact, even if their concentration in the marine bottom sediments generally reflected their concentration in the stream sediments, it was challenging to assess the real contribution of the many small and abandoned mine complexes, which behaved as a diffused source of contamination. Moreover, in a highly anthropised area such as the Entella basin, the impact of human activity is widespread both in the inland and directly on the coast. For example, the Zn concentration in the Entella Stream sediments seemed to suggest that a not negligible contribution of anthropic discharge exists, at least for this particular metal. Other elements seemed to derive from different sources. The sediment transport along the shore influenced the eastern sector of the Gulf of Tigullio. The input of the Gromolo Stream, characterised by the presence of metals related to the ophiolitic rocks (Cr, Mn, Pb, and partly V) and to the Libiola mine discharge (mainly Cu and Zn), partially overprinted the signal of the sediments transported by the Entella Stream.

Future chemical and mineralogical analyses of sediment sampled inside the two ports of Chiavari and Lavagna will help to verify the presence of metal enrichment points linked to human activities on the sea. Additional analysis of other factors, such as nutrient content in sediments, and additional sampling in different seasons could help to fully unravel the mechanisms that govern sediment distribution from the mouth of the Entella Stream and understand its dynamics also throughout the year and in different sea condition. A detailed research on sediments and flows of the stream during the year could also be useful to better understand the real amount of sediments involved on the continental shelf and their distribution area.
